# Final Validation of the Quality of Life after Brain Injury for Children and Adolescents (QOLIBRI-KID/ADO) Questionnaire

**DOI:** 10.3390/children11040438

**Published:** 2024-04-05

**Authors:** Nicole von Steinbuechel, Marina Zeldovich, Dagmar Timmermann, Ugne Krenz, Inga K. Koerte, Michaela V. Bonfert, Steffen Berweck, Matthias Kieslich, Marlene Henrich, Knut Brockmann, Anna Buchheim, Maike Roediger, Michael Lendt, Christian Auer, Axel Neu, Alexander Kaiser, Joenna Driemeyer, Sven Greving, Ulrike Wartemann, Daniel Pinggera, Claudius Thomé, Joachim Suss, Holger Muehlan, Katrin Cunitz

**Affiliations:** 1 Institute of Psychology, University of Innsbruck, Universitaetsstr. 5-7, 6020 Innsbruck, Austria; nicole.von-steinbuechel@uibk.ac.at (N.v.S.); marina.zeldovich@uibk.ac.at (M.Z.); anna.buchheim@uibk.ac.at (A.B.); 2 Faculty of Psychotherapy Science, Sigmund Freud University Vienna, 1020 Vienna, Austria; 3 Department of Psychosomatic Medicine and Psychotherapy, Division of Medical Psychology and Medical Sociology, University Medical Center Goettingen, Waldweg 37A, 37073 Goettingen, Germany; dagmar.timmermann@med.uni-goettingen.de; 4 University Medical Center Goettingen, Robert-Koch-Str. 40, 37075 Göttingen, Germany; ugne.krenz@web.de (U.K.); sven.greving@posteo.net (S.G.); 5 cBRAIN/Department of Child and Adolescent Psychiatry, Psychosomatics, and Psychotherapy, Ludwig-Maximilian University Hospital, LMU University, Nussbaumstrasse 5, 80336 Munich, Germany; inga.koerte@med.uni-muenchen.de; 6 Psychiatry Neuroimaging Laboratory, Department of Psychiatry, Mass General Brigham, 55 Fruit Street, Boston, MA 02114, USA; 7 Department of Pediatric Neurology and Developmental Medicine, LMU Center for Development and Children with Medical Complexity, Dr. Von Hauner Children’s Hospital, LMU University Hospital, Haydnstr. 5, 80336 Munich, Germany; michaela.bonfert@med.uni-muenchen.de; 8 Specialist Center for Paediatric Neurology, Neurorehabilitation and Epileptology, Schoen Klinik, Krankenhausstraße 20, 83569 Vogtareuth, Germany; sberweck@schoen-klinik.de; 9 Department of Paediatric Neurology, Hospital of Goethe University, Theodor-Stern-Kai 7, 60590 Frankfurt am Main, Germany; kieslich@med.uni-frankfurt.de (M.K.); marlene.henrich@kgu.de (M.H.); 10 Interdisciplinary Pediatric Center for Children with Developmental Disabilities and Severe Chronic Disorders, Department of Pediatrics and Adolescent Medicine, University Medical Center, Robert-Koch-Str. 40, 37075 Göttingen, Germany; knut.brockmann@med.uni-goettingen.de; 11 Department of Pediatrics and Adolescent Medicine, General Pediatrics, Intensive Care Medicine and Neonatology & Department of Pediatric Cardiology, University Hospital Muenster, Albert-Schweitzer-Campus 1, 48149 Muenster, Germany; maike.roediger@ukmuenster.de; 12 Neuropediatrics, St. Mauritius Therapeutic Clinic, Strümper Straße 111, 40670 Meerbusch, Germany; lendt@stmtk.de; 13 Johannes Kepler University Linz, Altenberger Straße 69, 4020 Linz, Austria; christian.auer2@kepleruniklinikum.at; 14 Department of Neurosurgery, Kepler Univesity Hospital GmbH, Wagner-Jauregg-Weg 15, 4020 Linz, Austria; 15 Department of Neurology and Neuropediatry, VAMED Klinik Geesthacht GmbH, Johannes-Ritter-Straße 100, 21502 Geesthacht, Germany; axel.neu@vamed-gesundheit.de (A.N.); alexander.kaiser@vamed-gesundheit.de (A.K.); 16 Department of Pediatrics, University of Hamburg-Eppendorf, Martinistrasse 52, 20246 Hamburg, Germany; j.driemeyer@uke.de; 17 Department of Neuropediatrics, VAMED Klinik Hohenstücken GmbH, Brahmsstraße 38, 14772 Brandenburg an der Havel, Germany; ulrike.wartemann@vamed-gesundheit.de; 18 Department of Neurosurgery, Tirol Kliniken GmbH, Medical University Innsbruck, Anichstrasse 35, 6020 Innsbruck, Austria; daniel.pinggera@tirol-kliniken.at (D.P.); claudius.thome@tirol-kliniken.at (C.T.); 19 Department of Pediatric Surgery, Wilhelmstift Catholic Children’s Hospital, Liliencronstraße 130, 22149 Hamburg, Germany; j.suss@kkh-wilhelmstift.de; 20 Department of Health and Prevention, University of Greifswald, Robert-Blum-Str. 13, 17487 Greifswald, Germany; holger.muehlan@uni-greifswald.de; 21 Department of Psychiatry and Psychotherapy, University Medical Center Goettingen, Von-Siebold-Str. 5, 37075 Goettingen, Germany

**Keywords:** health-related quality of life (HRQoL), pediatric traumatic brain injury (TBI), QOLIBRI-KID/ADO

## Abstract

Until recently, no disease-specific health-related quality of life (HRQoL) questionnaire existed for pediatric traumatic brain injuries (TBIs). In this revalidation study, the psychometric properties and the validity of the 35-item QOLIBRI-KID/ADO questionnaire in its final German version were examined in 300 children and adolescents. It is the first self-reported TBI-specific tool for measuring pediatric HRQoL in individuals aged between 8 and 17 years. The six-factor model fits the data adequately. The questionnaire’s internal consistency was excellent for the total score and satisfactory to excellent for the scale scores. Intraclass correlations indicated good test–retest reliability, and the measure’s construct validity was supported by the overlap between the QOLBRI-KID/ADO and the PedsQL, which measures generic HRQoL. The discriminant validity tests showed that older children and girls reported a significantly lower HRQoL than comparison groups, and this was also true of children who were anxious or depressed, or who suffered from post-concussion symptoms, replicating the results of the questionnaire’s first developmental study. Our results suggest that the QOLIBRI-KID/ADO is a reliable and valid multidimensional tool that can be used together with the adult version in clinical contexts and research to measure disease-specific HRQoL after pediatric TBI throughout a person’s life. This may help improve care, treatment, daily functioning, and HRQoL after TBI.

## 1. Introduction

Traumatic brain injuries (TBIs) in adolescence and childhood are one of the main causes of disability worldwide [1,2], affecting between 47 and 280 children and adolescents per 100,000 every year [3]. Following a TBI, impairment may persist for months or even years, affecting physical [4], cognitive [5], emotional [6], and social functioning [4]. These impairments may significantly impact quality of life (QoL). The World Health Organization defines QoL as “the individual’s perception of their position in life in the context of the culture and value systems in which they live and in relation to their goals, expectations, standards and concerns” [7]. Health-related quality of life (HRQoL), refers to an individual’s perception of their subjective physical and mental health, whereby a distinction is made between generic [8] and disease-specific [9] HRQoL. While generic HRQoL encompasses a wide spectrum of dimensions associated with different health conditions [8], disease-specific HRQoL looks at symptoms and situations that are specific to particular health conditions [9]. Thus, disease-specific measures of HRQoL are tailored and particularly sensitive to impairments resulting from a certain health condition and can more sensitively capture HRQoL in a patient population [10,11].

The recently developed Quality of Life after Brain Injury in Children and Adolescents (QOLIBRI-KID/ADO) questionnaire is the first self-reported, disease-specific HRQoL measure specifically designed for children and adolescents after a TBI aged between 8 and 17 years [12]. It was created using a specifically created item pool based on focus group interviews, national and international Delphi panels, adaptations of items from existing measures, and after cognitive debriefings. It was developed in the course of a German multicenter pilot study in 300 pediatric individuals who had suffered a TBI three months to ten years previously [12]. The QOLIBRI-KID/ADO consisted of 35 items covering six domains (i.e., Cognition, Self, Daily Life and Autonomy, Social Relationships, Emotions, and Physical Problems). The questionnaire was shown to be reliable and valid in capturing HRQoL in children and adolescents after a TBI [12,13]. Its psychometric properties were examined using methods referred to as the classical test theory (i.e., reliability and validity), supplemented by differential item functioning (DIF) analyses for the age groups. Satisfactory reliability was reported for both the total score (Cronbach’s α = 0.89) and the scales (Cronbach’s α = 0.70–0.80) in groups of children and adolescents with and without multiple symptoms [12]. It was also shown to have acceptable test–retest reliability (intraclass correlation coefficients: 0.42–0.64). Satisfactory construct validity was demonstrated compared to the Pediatric Quality of Life Inventory (PedsQL) [14] as a reference measure of generic HRQoL. Psychometric analyses using an item response theory framework indicated that the scales were largely unidimensional, there was no evidence to suggest that the assumptions of monotonicity and local independence were violated, and for the most part, no DIF occurred in the various patient groups [13].

Research on the impact of sociodemographic and clinical variables on HRQoL after a pediatric TBI is scarce. Reduced pediatric HRQoL has been found to be associated with older age [10,12], being female [15], suffering from adverse emotional states [12,16,17], a more severe TBI [18], a lower recovery [12,19], a larger number of post-concussion symptoms, and a comparatively recent TBI [12,17].

The present study aims to revalidate this 35-item questionnaire, with four items reworded, by replicating the structure and design of the developmental study in a different TBI sample. Several factors associated with impaired HRQoL after a pediatric TBI were examined to ensure the construct, discriminant, and convergent validity. We expected lower HRQoL to be associated with older age [20], being female [15], higher TBI severity [18,21], mental health issues—including anxiety [22] and depression [23]—as well as a larger number of post-concussion symptoms [17]. The final validation of the QOLIBRI-KID/ADO will support the assessment of its quality and its ability to capture the effects of pediatric TBI sequalae on children’s and adolescents’ HRQoL. We hope to improve diagnosis, treatment, monitoring, and research efforts to reduce the burden on children and adolescents after a TBI by providing a psychometrically robust instrument for measuring TBI-specific HRQoL.

## 2. Materials and Methods

### 2.1. Ethical Approval

This study was conducted in compliance with all the appropriate German and Austrian laws while taking into account the World Medical Association Declaration of Helsinki and the ICH Harmonized Tripartite Guideline for Good Clinical Practice. Each recruiting center gave this study ethical clearance, and all participants gave their informed consent in line with German and Austrian data protection laws. This study was approved by the Ethics Committee of the University Medical Center Goettingen (application no. 19/4/18).

### 2.2. Participants

**Figure 1:**
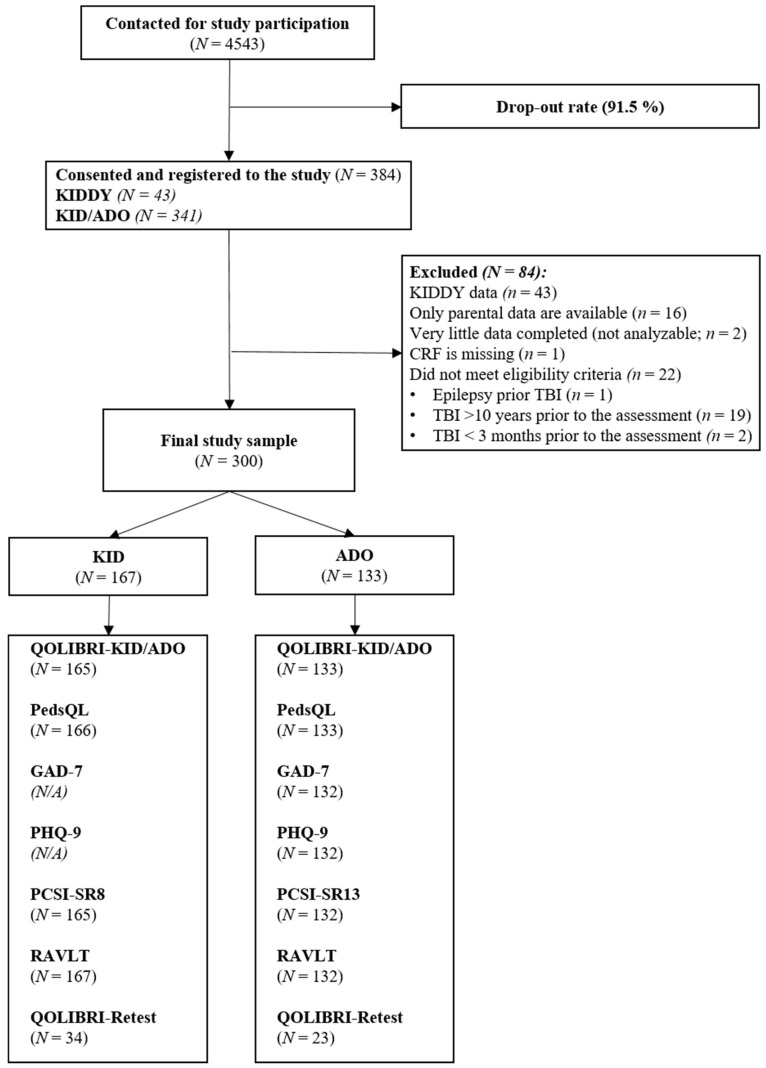
A flow chart for the number of participants for each instrument and the recruitment process. Note: TBI = traumatic brain injury; QOLIBRI-KID/ADO = Quality Of Life after Brain Injury in Children and Adolescents; PedsQL = Pediatric Quality of Life Inventory; GAD-7 = Generalized Anxiety Disorder 7; PHQ-9 = Patient Health Questionnaire 9 ; PCSI-SR8/13 = Postconcussion Symptom Inventory; RAVLT = Rey Auditory Verbal Learning Test.

For the statistical analyses, we met the requirements for the statistical power, which suggested a sample size of approximately 140 individuals per age group based on a simulation study [24]. Patients between the ages of 8 and 17 years at the time of joining the study were eligible to participate (February 2022 until February 2023). They also had to have been diagnosed with a TBI (at least three months but no more than ten years after the injury), be able to comprehend the questions and provide answers, and be outpatients (or have recently resumed inpatient treatment). Participants were excluded if they had any of the following criteria: a spinal cord injury, severe mental illness (such as psychosis or autism) or epilepsy prior to the TBI, lethal disease, very severe polytrauma, or no indication of the severity of their TBI. All legal guardians gave their written informed consent. The interviews were conducted face-to-face, either in person or online. If possible, interviews with children took place without the parents present to avoid possible influences. Written parental reports were requested by email, postal mail, or in person at the medical recruitment centers. Details of the sample composition are shown in Figure 1.

### 2.3. Sociodemographic and Clinical Data

The sociodemographic and clinical information was comparable to that collected in several QOLIBRI studies among adults [25,26] and the QOLIBRI-KID/ADO pilot study [12]. Parents provided information about themselves and their participating children, including education, occupation, gender, and age. 

Clinical data were collected from medical records, including loss of consciousness, presence of lesions according to imaging data (CT or MRI), the necessity for resuscitation, ventilation, or surgical interventions, post-traumatic epilepsy, and amnesia, as well as the cause, date, and severity of the TBI. The TBI’s severity (mild, moderate, severe) was derived either from the Glasgow Coma Scale (GCS) [27], the International Statistical Classification of Diseases and Related Health Problems (ICD-10) (diagnosis code: S06.*) [28], or from the clinical data on the TBI described above.

In addition, the medical records provided the time since the TBI and a median split of this time was used to construct participant subgroups. Further clinically relevant data were obtained from parental reports. Using a dichotomous scale (“Yes” or “No”), parents reported whether the children had mental health problems, headaches, other pain, or physical impairment following the TBI. The (mean) times since the TBI of participants with and without parent-reported problems, as well as those with mild to severe depression, anxiety, and self-reported above-average post-concussion symptoms, were compared (for more details, see the subsection “Instruments”).

### 2.4. Instruments

The QOLIBRI-KID/ADO questionnaire tested in the final validation study included 35 items assessing self-reported TBI-specific HRQoL in children and adolescents aged between 8 and 17 years on four “satisfaction” scales (“How satisfied are you with…”; dealing with cognitive abilities, self-concept, perceived autonomy, and social life) and two “bothered” scales (“How bothered are you with…”; describing physical and emotional problems). The responses were given on a five-point Likert-type scale from “Not at all” to “Very”, supplemented by smileys, using today and the past week as the time frame. Before being combined with the satisfaction scales for analysis, the responses to the two “bothered” scales had to be recoded in view of their negative wording. The responses for each QOLIBRI-KID/ADO scale were linearly transformed to values ranging from 0 to 100. Higher scores correspond to better HRQoL. Scale scores were calculated as the average of the item scores for each scale, and the total score was computed as the average of all the scales. Compared with the pilot study, four questions were rephrased, and misleading or unnecessary examples were removed to improve the comprehensibility of the items (see Appendix A, Table A1). 

The generic Pediatric Quality of Life Inventory (PedsQL™) [14] covers physical, social, emotional, and school functioning domains with 23 items. Scale scores can be aggregated to form a total, a physical, and a psychosocial health score. Participants were asked to answer using a five-point Likert-type scale from 0 to 4 (“Never” to “Almost always”). Responses were first inverted and then linearly transformed to a scale of 0 to 100. Higher scores indicate better HRQoL. 

Anxiety and depression symptoms were rated by parents using two screening instruments, the Generalized Anxiety Disorder 7 (GAD-7) scale [29] and the Patient Health Questionnaire 9 (PHQ-9) [30]. Clinical ratings could not be obtained because self-report questionnaires have not been validated for children under the age of twelve, so we applied the proxy-reports instead [31,32]. The GAD-7 [33] measures the occurrence of seven symptoms of anxiety in children and adolescents. Parents rated participants’ symptoms on a four-point Likert-type scale (“Not at all” (0) to “Nearly every day” (3)). The severity of the anxiety disorder is represented by the sum score of all the items, which ranges from 0 to 21: minimal (1–4), mild (5–9), moderate (11–14), and severe (15–21) anxiety symptoms [34]. The PHQ-9 uses DSM-IV [33] criteria to assess the presence and severity of symptoms related to major depression. Parents were asked to rate nine depression symptoms on a four-point Likert-type scale (“Not at all” (0) to “Nearly every day” (3)). The sum score of the items ranges from 0 to 27, leading to the following severity categories: minimal (1–4), mild (5–10), moderate (11–14), and severe (15–27) symptoms of depression [35]. 

Post-concussion symptoms were self-reported using the German version of the Postconcussion Symptom Inventory (PCSI-SR8 for children and PCSI-SR13 for adolescents) [36,37]. Eight to twelve-year-old children answered 16 items on a three-point Guttman scale (“No problem” (0) to “A lot of a problem” (3)); adolescents answered 21 items on a seven-point Guttman scale with three anchor categories (“Not a problem”, “Moderate problem”, and “Severe problem”). In both age versions, the items cover four domains (physical, emotional, cognitive, and sleep/fatigue); the sum across all the items represents the total score. Due to the lack of reference values, total scores are categorized as below, above, or within the average range (*M* ± 1 *SD*) of participants who completed the corresponding version of the PCSI. 

Using the King’s Outcome Scale for Closed Head Injury (KOSCHI) [38], examiners and clinicians rated functional recovery/disability after a TBI using the following categories: 3a: “lower severe disability”, 3b: “upper severe disability”, 4a: “lower moderate disability”, 4b: “upper moderate disability”, 5a: “good recovery”, and 5b: “intact recovery”. 

The Rey Auditory Verbal Learning Test (RAVLT) [39,40] was used to measure verbal learning and memory. Fifteen words were read aloud by the examiners, and these had to be repeated by the children in eight trials. The number of words recalled in Trial I was subtracted from the number in Trial V to determine the learning rate, which was divided into three categories: above, below, or within the age group’s typical range (*M* ± 1 *SD*).

### 2.5. Statistical Analyses

Descriptive statistics at the item, scale, and total score levels included means (*M*), standard deviations (*SD*), skewness (*SK*), and floor and ceiling effects. Skewness was considered absent for values from −0.5 to 0.5; values from ±0.5 to ±1 were considered moderate, and values beyond ±1 were heavily skewed [41]. The percentage of responses in the most satisfied/least bothered category was used to report ceiling effects, while the percentage in the least satisfied/most bothered category indicated floor effects [42]. 

Differential item functioning (DIF) analyses were used to investigate whether participants’ responses to the final version of the QOLIBRI-KID/ADO—were independent of their age. We analyzed this by calculating the logistic ordinal regression of differential item functioning (LORDIF) [43] based on the age category. Each item was treated as a dependent variable, and the DIF was evaluated by comparing a LORDIF model, including the scale scores, with a model including the scale scores, age category, and an age category–scale score interaction. Whenever the comparison of the models revealed a significant difference (α = 0.01) and the associated effect exceeded a very small effect (McFadden’s pseudo R^2^ > 0.05) [44], the response behavior was assumed to differ between age categories.

The reliability of the QOLIBRI-KID/ADO was analyzed by testing for internal consistency and test–retest reliability. Cronbach’s α and McDonald’s ω coefficients were calculated to assess the internal consistency of the total and scale scores. In line with findings from pediatric outcome research, Cronbach’s α ≥ 0.60 [8] and McDonald’s ω ≥ 0.70 [45] were regarded as satisfactory. It has been reasoned that the internal consistency of HRQoL measures may be lower in individuals with cognitive impairment [12,46]. We, therefore, conducted subgroup analyses based on the RAVLT learning rate. To examine the reliability of the questionnaire in more detail for different TBI-related symptoms, its internal consistency was calculated and stratified by severity and symptom groups (for a list and classification, see the subsection “Computational Software, Classification, and Cut-Off Values”). In addition, corrected item–total correlations (CITC) were calculated for individual items and their corresponding scales, with a criterion of CITC > 0.40. Test–retest reliability was determined using a subsample of participants who completed the QOLIBRI-KID/ADO twice, with an interval of 10 to 20 days between each assessment. Intraclass correlation coefficient (ICC) analyses were carried out based on a two-way random effects model for the total score and each scale. ICC values > 0.60 [47] were considered satisfactory. In addition, the standard error of measurement (SEm) [48] and the minimal detectable change (MDC) were calculated based on the ICC of the total and scale scores. Since there are no clear criteria for acceptable MDC and SEm values, SEm was considered in terms of the total possible uncertainty range of the instrument. A percentage less than 10%, corresponding to a variation of 10 points on the 0 to 100 scale score, was considered satisfactory [49].

The QOLIBRI-KID/ADO questionnaire’s factorial structure was analyzed through confirmatory factor analysis (CFA). A six-factor model was assumed, representing the six scales of the QOLIBRI-KID/ADO. Given the ordinal nature of the response categories, diagonally weighted least squares with mean and variance adjustment (WLSMV) were used as the estimate for model identification [50]. As the cut-off values were established for estimating maximum likelihood and not for the estimation method, the following cut-offs for the model fit indices must be interpreted with caution [51]. The indices used, with their respective desirable cut-offs in parentheses, were χ^2^-test statistics (*p* > 0.01) [52], χ^2^/df ratio (≤2) [52], comparative fit index (CFI > 0.95) [53,54], Tucker–Lewis index (TLI > 0.95) [54,55], root mean square error of approximation and its 90-percent confidence interval (RMSEA < 0.06, CI90%) [56], and the standardized root mean square residual (SRMR < 0.08) [54]. 

To examine whether the QOLIBRI-KID/ADO questionnaire’s final version measures the HRQoL construct, its convergent validity was investigated against the PedsQL, an established pediatric measure of generic HRQoL. This involved the computation of Pearson correlation coefficients to examine the relationship between all scales: the QOLIBRI-KID/ADO total score, the physical functioning score (equal to the Physical Problems scale), and the psychosocial functioning score (mean of Cognition, Self, Social Relationships, and Emotions scales) with the corresponding PedsQL scale scores. 

Discriminant validity was determined by analyzing the correlations with constructs presumed to be less closely related. The association between the construct and relevant sociodemographic and clinical characteristics was investigated using correlational and known-group analyses [57]. The associations between the QOLIBRI-KID/ADO total and scale scores to the PCSI-SR8/13, GAD-7, and PHQ-9 scores, were inspected by means of their Pearson correlation coefficients. The GAD-7, PHQ-9, and PCSI-SR8/13 were expected to display negative correlations corresponding to a small to medium effect (i.e., r between −0.10 and −0.30) [58], demonstrating a degree of overlap across the domains and pointing to a possible relationship between lower HRQoL and increased symptom burden. To determine the amount of variance explained, the coefficient of determination (R²) was calculated.

### 2.6. Missing Values 

The occurrence of missing values was not considered a problem if missing values did not exceed five percent of the responses to an item. Total scores were calculated if all scale scores were present. The QOLIBRI-KID/ADO scale scores were calculated if no more than one-third of a scale’s responses were absent. Following the manual of the PedsQL [59], mean scale scores were computed as long as no more than half of a scale’s responses were missing. Similarly, the mean scores for the GAD-7 and PHQ-9 and the sum score for the PCSI-SR8/13 were calculated, provided that the number of items missing on each measure was no more than one-third.

### 2.7. Computational Software, Classification, and Cut-Off Values 

The software R (version 4.3.0) [60] was used for all the analyses, using the following packages: psych [61] for calculating reliability measures, lavaan [62] for the CFA, and lordif [63] for DIF analyses. 

The following TBI-related symptoms were considered when analyzing the symptom burden: low functional recovery (expressed as a KOSCHI score below 5), parent-reported post-TBI problems (headache or other aches, physical impairments, or mental health problems: if at least one is reported by a parent), symptoms of depression and anxiety (expressed by PHQ-9 and GAD-7 scale scores above 4, respectively), post-concussion symptoms (expressed as PCSI-SR8/13 scale scores greater than or equal to 1 SD above the age-adjusted mean), and a low learning rate (expressed as corresponding RAVLT scores less than or equal to 1 SD below the age-adjusted mean). The symptom burden was examined for the total sample and the different TBI severity levels (mild vs. moderate/severe). 

Cohen’s cut-off criteria were used to classify the strength of these associations, indicating a small (0.10), moderate (0.30), or large (0.50) effect [58]. For the comparisons presented, moderate correlations (r ≥ 0.30) were required in order to confirm convergent validity; small to medium correlations were expected to indicate sufficient discrimination between the constructs. The mean comparisons for the known-groups validity analyses used one-tailed Student’s *t*-tests. The correlation analyses used Pearson correlation coefficients. 

For statistical comparisons, *p*-values below 0.05 were considered significant for the total score. For the scale comparisons, *p*-values were adjusted using the Bonferroni correction (i.e., 0.05/6 = 0.008).

## 3. Results

### 3.1. Participants

The final version of the QOLIBRI-KID/ADO was completed by 300 children and adolescents. Data from 41 participants had to be excluded because only parental data were available, insufficient data were provided, or inclusion criteria had been violated (one participant was diagnosed with epilepsy prior to their TBI). Twenty-two participants completed the questionnaire outside the allotted time frame of 3 months to 10 years after their TBI. 

Participants were interviewed face-to-face, either online (60%) or in person (40%). Compared with participants who were interviewed in person, those who took part online had higher QOLIBRI-KID/ADO total scores (*M_online_* = 75.67 vs. *M_in-person_* = 79.49, t(296) = −2.73, *p* = 0.005, *d* = 0.33). Ninety-four (31%) participants had also taken part in the pilot study; however, prior participation had no effect on the QOLIBRI-KID/ADO total scores in the present study (*M_repeated_* = 77.78 vs. *M_naïve_* = 76.95, t(296) = 0.56, *p* = 0.574, *d* = 0.07). 

Table 1 summarizes the sociodemographic and clinical data. The mean age was 12.6 years (*SD* = 2.67, *Min* = 8.00, *Max* = 17.90), and 54% were male. Mild TBI was the most common type of severity (80%), of which 21 participants (9%) sustained complicated mild TBIs [64] (i.e., mild TBIs with brain lesions identified via a CT or MRI). Overall, lesions were present in 24% of all participants. The most common onset of a TBI was four to ten years prior to study enrollment (67%). The majority of participants displayed favorable functionality (5a/b) based on their KOSCHI score (94%). The parents of half the participants (49%) reported their children as having post-TBI problems. Participants’ self-rated post-concussion symptoms were mostly within the average range for their age group (75%). Most participants showed no or minimal signs of anxiety (71%) or depression (70%). We also examined the symptom characteristics of participants whose parents reported that they had mental health problems, headaches or other pain, some physical impairment, mild to severe anxiety or depression, and a larger number of post-concussion symptoms in relation to the time since their TBI. Only participants with parent-reported problems after their TBI differed significantly from those without, with a shorter mean time since their injury (*M_problem_s* = 5.06, *M_no problems_* = 5.71, t(296) = 2.09, *p* = 0.038, *d* = 0.24; see Appendix A, Table A2).

### 3.2. Item Analyses of the Final QOLIBRI-KID/ADO Questionnaire: Item Properties, Internal Consistency, and Differential Item Functioning

The item characteristics and distributions are shown in Table 2. Given the low number of missing values per item (≤1%), we assumed these rare cases would not affect the assumption that missingness is completely random. The means of all items were above three on the five-point Likert-type scale, and all items were skewed to the left. Most responses also displayed ceiling effects, a common observation in this kind of HRQoL assessment [65].

Regarding the contribution of individual items to internal consistency, two items from the Cognition scale (“Decision between two things” and “Orientation”) had corrected item–total correlations below 0.40. The item “Loneliness” from the Emotion scale also led to a slight increase (+0.01) in Cronbach’s α coefficient when omitted (see Table 2). 

The DIF analysis did not reveal any meaningful differences between children and adolescents in the response pattern for individual items. Although three items (“Planning”, ”Sadness”, and “Other injuries”) showed significant differences when comparing models with and without assumed age-group effects, the differences found were not sufficiently large to be considered to have an impact. For details, see the right-hand side of Table 2.

The internal consistency of the total score of the QOLIBRI-KID/ADO was excellent, with Cronbach’s α = 0.92 and McDonald’s ω = 0.92. The individual QOLIBRI-KID/ADO scales had good to excellent internal consistencies (Cronbach’s α: 0.69–0.77; McDonald’s ω: 0.76–0.85; see Table 2 for details), which matches the findings from the pilot study. Subgroup analyses of the internal consistencies for individual scales and subgroups with lower learning rates showed, for the most part, satisfactory to excellent results (Cronbach’s α: 0.65–0.85; McDonald’s ω: 0.76–0.89). This was also the case for other subgroups stratified by sociodemographic and clinical characteristics (Cronbach’s α: 0.87–0.94; McDonald’s ω: 0.89–0.96), except for the group with present post-concussion symptoms on the Cognition scale (Cronbach’s α = 0.52). For details, see Appendix A, Table A3.

### 3.3. Scale Analyses of the QOLIBRI-KID/ADO Questionnaire: Descriptive Statistics, Test–Retest Reliability, and Factorial Validity 

Table 3 summarizes the descriptive statistics for the QOLIBRI-KID/ADO, PedsQL, PHQ-9, GAD-7, and PCSI-SR8/13. The mean scale scores for the QOLIBRI-KID/ADO were higher than 60, as is often observed in HRQoL studies [65], especially in children and adolescents [66]. Almost all the scales of the QOLIBRI-KID/ADO and the PedsQL were skewed to the left, except for the Cognition and Emotions scales of the QOLIBRI-KID/ADO, which only showed a slight tendency to be skewed in this direction. On average, the levels of anxiety, depression, and post-concussion symptoms reported were low.

For the test–retest analysis, 57 participants completed the QOLIBRI-KID/ADO questionnaire twice after a specified delay of 10–20 days. The individual scale ICCs ranged from 0.57 to 0.74 and the total scale ICC was 0.78; thus, almost all ICCs were above the 0.60 criterion, except for the Physical Problems scale (see Table 4 for details). Expressed as a percentage of the measure’s total possible range, almost all the SEm values remained below the 10% criterion. The MDC of the total score and the scales ranged from 13.35 to 34.91.

**Figure 2:**
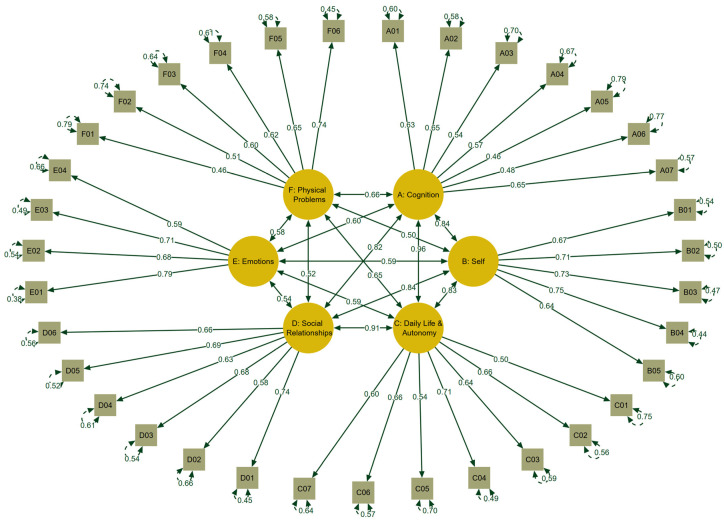
Results of the confirmatory factor analysis of the QOLIBRI-KID/ADO questionnaire. Note: Standardized coefficients—A1–7: Cognition (six items: Concentration, Talking to Others, Remembering, Planning, Decision Between Two Things, Orientation, and Thinking Speed); B1–5: Self (five items: Energy, Accomplishment, Appearance, Self-Esteem, and Future); C1–7: Daily Life and Autonomy (seven items: Daily Independence, Getting out and About, Manage at School, Social Activities, Decision Making, Support from Others, and Ability to Move); D1–6: Social Relationships (six items: Open up to Others, Family Relationship, Relationship with Friends, Attitudes of Others, and Demands from Others); E1–4: Emotions (four items: Loneliness, Anxiety, Sadness, and Anger); and F1–6: Physical Problems (six items: Clumsiness, Other Injuries, Headaches, Pain, Seeing/Hearing, and TBI Effects).

Looking at the factorial validity, it was found that the lowest response categories were rarely chosen; this made it necessary to collapse the response categories “not at all” and “slightly” for the “satisfaction items” as well as “very” and “quite” for the “bothered scales” for the CFA analyses. The six-factor model produced fit indices that fulfill the requirements for an excellent fit (CFI = 0.99, TLI = 0.99, RMSEA = 0.03, CI_90%_ [0.03, 0.04], SRMR = 0.07), the sole exception being the χ²(545) = 729.20 index (*p* < 0.01). The significant *p*-value is attributable to the large number of degrees of freedom (545). However, the ratio between χ² and df was 1.34, i.e., below the acceptable cut-off of 2. These findings match those obtained during the pilot study. The resulting factor loadings are shown in Figure 2.

**Figure 3:**
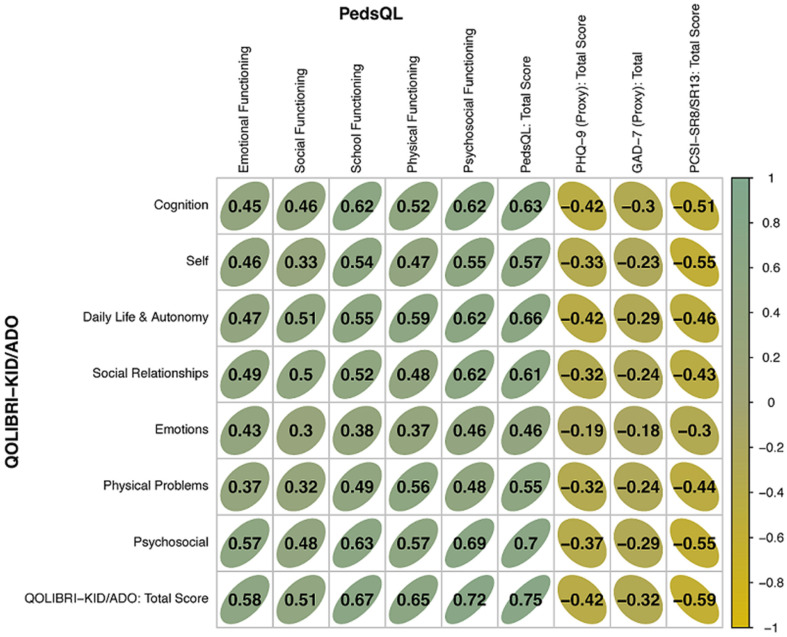
Correlation results of convergent and divergent validity analyses (Pearson correlation coefficients).

Figure 3 summarizes the Pearson correlation coefficients for the construct validity, and Appendix A, Table A4, shows the corresponding explained variance obtained from correlating the QOLIBRI-KID/ADO total and scale scores with the PedsQL total and scale scores and with the total scores of the GAD-7, the PHQ-9, and the PCSI-SR8/13. The constructs of the QOLIBRI-KID/ADO and PedsQL that were compared included the emotional, social, physical, and psychosocial dimensions, and the total scores. Correlations between the QOLIBRI-KID/ADO and PedsQL scales ranged from *r* = 0.43 (Emotions and Emotional Functioning), *r* = 0.5 (Social Relationships and Social Functioning), *r* = 0.56 (Physical Problems and Physical Functioning), *r* = 0.69 (Psychosocial Functioning scale scores) to *r* = 0.75 (total scores of the QOLIBRI-KID/ADO and PedsQL). The corresponding explained variances were between 18% (emotional), 25% (social), 31% (physical), 48% (psychosocial), and 56% (total), supporting construct validity.

The divergent validity analyses, tested against instruments measuring anxiety, depression, and post-concussion symptoms, revealed the hypothesized negative correlations. The QOLIBRI-KID/ADO scales ranged between r = −0.42 and r = −0.18 against the GAD-7 and PHQ-9 and between r = −0.3 and r = −0.55 against the PCSI-SR8/13, explaining 3% to 18% of the variability in the scores. The correlations of the total scores were r = −0.42, r = −0.32, and r = −0.59, with explained variances of 17%, 10%, and 34%, respectively, indicating that the QOLIBRI-KID/ADO questionnaire has good discriminant validity when compared with the measures for emotional states and post-concussion symptoms.

Table 5 summarizes the results of known group comparisons that were hypothesized to differ in TBI-related HRQoL, providing further indication of construct validity. Children were found to have significantly higher QOLIBRI-KID/ADO total scores than adolescents. Looking more closely at the scales for which children reported higher HRQoL than adolescents, a significant difference was found for the Self scale (*t*(296) = −5.21, *p* < 0.001, *d* = −0.61). The total scores of the QOLIBRI-KID/ADO were significantly higher for male participants than female participants. Looking at the individual scales on which males reported higher HRQoL than females, a significant difference was also found on the Self scale (*t*(296) = −2.89, *p* < 0.001, *d* = −0.34).

In terms of TBI-related characteristics, the QOLIBRI-KID/ADO total and scale scores were not affected by the time since the TBI or by the TBI severity (*p* > 0.05). Participants without post-concussion symptoms had significantly higher scores on the QOLIBRI-KID/ADO total (*p* < 0.05) and on all individual scales (p_adjusted_ < 0.008) than those with symptoms. Similarly, participants whose parents had reported no headaches, other pain, physical impairment, or mental health problems after their TBI had significantly higher QOLIBRI-KID/ADO total and Cognition and Physical Problems scale scores than participants whose parents reported these problems. Furthermore, participants with a low learning rate did not differ significantly from participants with an average to high learning rate with respect to their QOLIBRI-KID/ADO scores. Regarding emotional states, the absence of symptoms of depression or anxiety was associated with higher QOLIBRI-KID/ADO total and scale scores. 

## 4. Discussion

The aim of this study was to develop the first disease-specific tool for assessing HRQoL, specifically in children and adolescents after a TBI. Our results indicate that the final version of the QOLIBRI-KID/ADO questionnaire is a valid and reliable pediatric TBI-specific HRQoL measure that can be used in research, diagnostic, and therapeutic contexts. Our study can be seen as complementary to face-to-face interviews performed in clinical settings. Especially from the age of 12 years onwards, HRQoL should possibly be self-reported as this best reflects the concept. The questionnaire addresses six relevant dimensions of life that could be crucial for children and adolescents who have experienced a TBI [67]: Cognition, Self, Daily Life and Autonomy, Social Relationships, Emotions, and Physical Problems.

The six subscales displayed satisfactory to good internal consistency, whereas the total score even achieved excellent internal consistency values. The internal consistency was also found to be largely sufficient and satisfactory for different subgroups of children/adolescents with symptoms of anxiety or depression or with lower learning rates. This supports the use of the questionnaire in populations with different symptom levels, potentially impacting the evaluation of HRQoL in diagnostic and therapeutic processes [68]. 

Furthermore, the revalidation of the QOLIBRI-KID/ADO questionnaire confirms that it is a reliable, valid, and stable measure over the test–retest interval. The total score displayed very good reliability, as indicated by the ICC, and the scales demonstrated a fair to good agreement between test and retest [47]. The SEm values of less than 10% for the total and all scale scores, apart from the Emotions and Physical Problems scales, reflect some variability in the data. The MDC values indicate that a change of more than 13 points in the QOLIBRI-KID/ADO total score and a change of 15 points or more in the scale scores can be considered a “true” change [49] in disease-specific HRQoL after a TBI. Longitudinal studies on treatment or therapy are recommended to address the QOLIBRI-KID/ADO’s sensitivity to change and meaningful clinical change in more detail.

The factorial validity analyses replicated the one-level six-factor structure with excellent fit indices, comparable to the pilot study’s findings [12] and the adult QOLIBRI assessment [25]. The one-level six-factor structure supports the notion that the six factors of the multidimensional HRQoL construct should also be considered in research and clinical practice [69].

Using the same questionnaire for 8 to 17-year-olds is supported by the lack of meaningful item differences between the two age groups. This allows aggregated analyses and long-term follow-ups using the QOLIBRI-KID/ADO and underlines that the questionnaire is suitable for comparing these age groups.

The majority of the participants expressed comparatively high satisfaction with their HRQoL in terms of Cognition, Self, Daily Life and Autonomy, and Social Relationships and did not report feeling bothered much by Emotions or Physical problems (QOLIBRI-KID/ADO scores > 62). This may be partially explained by the composition of the study sample. Other studies have reported a decrease in HRQoL in individuals after moderate-to-severe TBIs [18], with chronic medical disorders [70], or with cognitive impairments [71]. In our sample, symptoms of anxiety or depression were reported for more than 30% of the participants; 19% had a low learning rate, and 18% suffered from post-concussion symptoms. Furthermore, half of the parents described their children/adolescents as having experienced at least one issue following the TBI: mental health problems, headaches, other pain, or physical impairment.

We observed an unexpected difference in the total score between those tested online and those tested in person. There was a small effect with a significantly lower HRQoL in the online group, which can be attributed to the fact that the online group included more participants who had experienced a TBI within the first year (8.4% vs. 0%) and had a higher incidence of moderate-to-severe TBIs (23.4% vs. 14.8%) compared with those tested face-to-face. Previous study participation (31.3%) had no significant effect on HRQoL.

We were able to replicate all the results of the previous pilot study regarding the validity of the QOLIBRI-KID/ADO. Evidence of construct validity was obtained by comparing the total score, the physical functioning, and the psychosocial functioning scores with the corresponding scales of the generic HRQoL measure, the PedsQL. The correlations calculated for the total scores and both physical and psychosocial functioning scales were fairly high, explaining up to 56% of the variance and supporting the construct validity of the new measure. The correlations between the total score of the QOLIBRI-KID/ADO and the symptoms of anxiety and depression were moderate and negative, in line with the correlations found between these concepts in other studies [22,23]. This suggests that lower levels of HRQoL correspond to higher levels of symptom burden and vice versa. However, the overlap between the constructs suggested by the explained variance is relatively low, which was also hypothesized in terms of the divergent validity. Previous research has found that 11 to 45% of children risk developing mental health issues following a pediatric TBI [72]. In our study, even several years after their TBIs, one-third of participants experienced mild-to-severe symptoms of anxiety and depression, with a notable decline in HRQoL. Similar mental health outcomes have also been reported for children and adolescents coping with chronic health conditions [16]. By identifying emotional problems early on, the rehabilitation of the children and adolescents concerned, as well as their families, could be improved since the negative effects of their TBI, including mental health problems, may only manifest several years after the injury. From pediatric TBI review studies, it is known that several factors may influence HRQoL [18]. As in the developmental pilot study [12], the current study also found higher total HRQoL scores, especially higher scores on the Self scale, in children than in adolescents. Other pediatric studies have also reported an increase in health complaints [73,74] and a decrease in HRQoL with increasing age [20,75,76,77,78]. This may be explained by the fact that adolescence and puberty are periods of major developmental, physical, and social transitions due to the maturation process [79], where individuals often face challenges in coping with their environment [80]. Also, younger children may endure fewer dramatic life changes after a TBI simply because they may have limited memories of their lives before the TBI occurred [23].

Our sample included only slightly more boys (54%), which is relatively more balanced in terms of sex compared with other QOLIBRI studies [12,26,81]. In general, males have a higher incidence of TBIs [82] and report fewer health complaints than females, at least in industrialized countries [73,83], resulting in a higher HRQoL [15]. Accordingly, and as in the pilot study [12], boys reported a significantly higher total HRQoL; the Self scale, particularly, was rated more positively than among girls. This may be due to a conglomerate of genetic, cultural, and social factors [83]. Different societal expectations [76] regarding roles and positions [84] may explain why girls, in general, have a less optimistic view of happiness [78] and are generally more worried [83]. They tend to internalize problems and negative emotions [85], which makes them more vulnerable to later mental health problems [85]. They may, therefore, benefit from engaging in greater reflection and more communication about their feelings [78]. 

As observed in previous research [18] and in our pilot study [12], the current study found no link between moderate-to-severe TBIs and decreased HRQoL, possibly due to the non-linearity of the TBI severity and HRQoL [86]. We discovered no significant relationship between HRQoL and the length of time since the TBI, in contrast to other studies that suggest that longer times since the TBI are linked to better HRQoL outcomes [23]. However, when the TBI occurred more recently, we observed a negative effect on HRQoL in those with more post-concussion and depression symptoms. This contrasts with other research, which suggests that persistent symptoms and adverse outcomes following early pediatric TBIs may not manifest until later in life [87]. The symptoms reported in our study several years after TBIs were only minimal. This may be due to the predominantly mild severity of the TBIs experienced by our study participants. Previous studies have suggested that the association between symptom duration and TBI severity may be stronger than it is with the time since the TBI or the age of the child [88]. This issue should be explored further in future studies by additionally controlling potential confounders [89] (e.g., other health conditions, comorbidities, or also considering developmental stages leading to depressive symptoms) that may affect adolescents between the time of injury and the assessment of post-concussion, depression symptoms, and HRQoL beyond the acute phase of injury.

In line with the pilot study [12] and other research findings [23,90], we found small but significant effects indicating that more post-concussion, anxiety, and depression symptoms, as well as parent-reported problems, are associated with lower HRQoL. Children who have suffered a TBI are generally more likely to experience anxiety [91], depression [72], and persistent post-concussion symptoms [17,92]. It is known from the adult literature [93] that the persistence of symptoms and emotional states compromise HRQoL even years after a TBI. The rehabilitation and HRQoL of children and adolescents following a TBI might improve if emotional difficulties can be identified and treated earlier on. 

Overall, we were able to replicate the robust psychometric structure found in the developmental study, as well as the construct validity and the significant impact of age, sex, anxiety, depression, and post-concussion symptoms on HRQoL assessed using the first disease-specific QOLIBRI-KID/ADO after a pediatric TBI. It is important to note that severity did not affect HRQoL in either study, but caution should be exercised in interpreting this finding due to the characteristics of the sample, with children and adolescents predominantly having a mild TBI.

### Limitations 

This study complements the development and validation of the QOLIBRI-KID/ADO [12], the first disease-specific questionnaire to assess HRQoL after a pediatric TBI. However, some limitations should be mentioned. Concerning the study sample, less than 10% of those originally contacted decided to participate in this study. The reasons for non-participation in pediatric clinical studies are formally unknown, but a self-selection bias [94] may potentially explain the high drop-out rate observed. Reasons for non-participation may involve parental concerns. Several parents expressed worries that their children were too healthy to participate or might not remember the accident, while others were extremely concerned that their children could be retraumatized by participating in this study, as mentioned in other studies [95,96].

Furthermore, like in many observational studies on TBIs [18], most participants had experienced a mild TBI, and only in 24% of cases were lesions found in imaging. This reflects the epidemiology of the prevalence of pediatric TBIs, with 91 to 97% of individuals with mild TBIs in Germany [82]. Our sample’s probability of pathological morphological findings corresponds to the determined GCS values. Lesions can be expected in 6.0% of the concerned individuals with a GCS of 15 and 18.9% of those with a GCS of 14 [82]. In addition, the injury dates back four to ten years in most cases. Because of recruitment difficulties and to represent a great variety of patients in the time since the injury, thus enhancing the validation quality, we used a time window from 3 months to 10 years after the TBI. Therefore, in this study, the long-term effects of TBI could only be partially assessed. For the long-term effects of TBIs over the lifespan, the adult version of the QOLIBRI questionnaire could be applied with a validated age range from 18 to over 80 years. The sample composition may, therefore, explain why the lower response categories, indicating low satisfaction, were rarely selected by participants. This, in turn, caused some problems with model estimation, which were solved by subsequently merging the two lower response categories. This limited variability in the sample could pose challenges in interpreting our results and may reduce their generalizability. However, we found that time since the TBI, but not TBI severity, had an impact on HRQoL, as measured by the QOLIBRI-KID/ADO total score. Since our tool is able to identify HRQoL differences even amongst individuals who experienced a mild TBI, we believe that the instrument may be able to differentiate HRQoL in patients who have experienced a moderate-to-severe TBI.

A potential limitation may also be the use of parent ratings to assess depressive and anxious symptoms in children and adolescents. Due to a lack of validated screening instruments for anxiety/depression in the young pediatric population, we used parent reports. However, discrepancies in agreement between self-reports and parent ratings, particularly in younger children, may limit the validity of parent ratings as proxies for child self-reports [97,98]. In view of this known limited parent–child agreement, we strongly recommend that future research develops and validates screening tools for the pediatric population after TBIs, which can be used as self-reports.

In order to address the lack of studies and the limitations of clinical, pediatric research on the long-term effects of TBIs on HRQoL, as well as the small (sub-)sample sizes and generalizability that are typical of clinical single-center studies, we recommend organizing large international studies similar to the observational EU Center TBI study in adults after a TBI as a way of filling in some of the knowledge gaps mentioned. 

## 5. Conclusions

The QOLIBRI-KID/ADO questionnaire is the first validated, disease-specific instrument for measuring disease-specific HRQoL after a pediatric TBI. The psychometric properties of this comprehensive 35-item self-report instrument demonstrate the necessary feasibility and measurement characteristics in six dimensions of HRQoL in children and adolescents after a TBI. The QOLIBRI-KID/ADO successfully captures the expected theoretical links between the construct of HRQoL and other constructs and aspects, thereby supporting its construct validity. Our findings emphasize the importance of implementing prevention strategies that are sensitive to age and sex differences, as well as to anxiety, depression, and other symptoms, such as post-concussion or cognitive symptoms, which occur after a pediatric TBI. Therefore, the QOLIBRI-KID/ADO could be applied in research and integrated into healthcare protocols to create rehabilitation circulars for planning and ameliorating interventions and subsequent outcomes after a TBI.

## Figures and Tables

**Table 1:** Descriptive statistics on the sociodemographic and clinical characteristics of the final validation study participants.

Variable	Group	N (%)
Sex	Female	137 (46%)
	Male	163 (54%)
Age	Children (8–12 years)	167 (56%)
	Adolescents (13–17 years)	133 (44%)
Parents’ Highest Education	Primary school	1 (0%)
	Secondary school	51 (17%)
	Vocational school	54 (18%)
	College/university	193 (64%)
	Unknown or missing	1 (0%)
TBI Severity	Mild	240 (80%)
	Moderate	30 (10%)
	Severe	30 (10%)
Cerebral Lesion(s) Found in Neuroimaging	No	227 (76%)
	Yes	73 (24%)
KOSCHI Disability/Recovery Score	4b	15 (5%)
	5a	21 (7%)
	5b	261 (87%)
	Missing	3 (1%)
Time Since TBI	<1 Year	15 (5%)
	1–<2 Years	30 (10%)
	2–<4 Years	55 (18%)
	4–10 Years	200 (67%)
Mental Health Problems, Head-/aches, or Physical Impairments after TBI	No	151 (50%)
	Yes	147 (49%)
	Missing	2 (1%)
Learning Rate (RAVLT)	Below average (≤*M* − 1*SD*)	57 (19%)
	Average	177 (59%)
	Above average (≥*M* + 1*SD*)	65 (22%)
	Missing	1 (0%)
GAD-7	No anxiety (0)	46 (15%)
	Minimal anxiety (1−4)	166 (55%)
	Mild to severe anxiety (>4)	87 (29%)
	Missing	1 (0%)
PHQ-9	No depression (0)	34 (11%)
	Minimal depression (1−4)	176 (59%)
	Mild to severe depression (≥5)	89 (30%)
	Missing	1 (0%)
PCSI-SR8/13	Below average (≤*M* – 1*SD*)	26 (9%)
	Average	224 (75%)
	Above average (≥*M* + 1*SD*)	47 (16%)
	Missing	3 (1%)

Note: TBI = traumatic brain injury; KOSCHI = King’s Outcome Scale for Closed Head Injury; RAVLT = Rey Auditory Verbal Learning Test; GAD-7 = Generalized Anxiety Disorder 7 (proxy report); PHQ-9 = Patient Health Questionnaire 9(proxy report); PCSI-SR8/13 = Postconcussion Symptom Inventory (age adapted *M/SD*; self-report); *M* = mean; *SD* = standard deviation.

**Table 2:** Item and scale statistics of the QOLIBRI-KID/ADO questionnaire: descriptive statistics, internal consistency parameters, and DIF analyses.

			Descriptive Statistics						Internal Consistency			*CITC*	DIF Analyses	
Scale	Item Abbreviation	Items	*M*	*SD*	% Missing	*SK*	% Floor	% Ceiling	α	Changes in α if Omitted	ω		*p*	*R*²
Cognition									0.69		0.79			
	A1	Concentration	3.91	0.85	1	−0.63	5	72		−0.07		0.64	0.754	
	A2	Talking to Others	4.45	0.69	0	−0.99	1	90		−0.03		0.46	0.153	
	A3	Remembering	3.91	0.92	1	−0.57	8	70		−0.03		0.49	0.035	
	A4	Planning	4.06	0.96	0	−0.90	7	75		−0.04		0.51	0.003 *	0.015
	A5	Decision Between Two Things	3.45	1.06	0	−0.41	17	51		−0.01		0.37	0.016	
	A6	Orientation	4.79	0.49	0	−2.70	1	97		0.00		0.27	0.212	
	A7	Thinking Speed	3.97	0.81	0	−0.55	3	74		−0.08		0.70	0.164	
Self									0.76		0.81			
	B1	Energy	3.95	0.94	0	−0.88	8	74		−0.03		0.58	0.815	
	B2	Accomplishment	4.07	0.69	0	−0.45	2	83		−0.03		0.58	0.820	
	B3	Appearance	4.12	0.85	1	−0.88	4	78		−0.08		0.71	0.216	
	B4	Self-Esteem	4.26	0.87	1	−1.16	3	82		−0.06		0.67	0.098	
	B5	Future	4.13	0.9	1	−0.94	5	78		−0.02		0.53	0.163	
Daily Life and Autonomy									0.73		0.80			
	C1	Daily Independence	4.76	0.51	0	−2.17	0	97		−0.02		0.47	0.096	
	C2	Getting Out and About	4.64	0.62	0	−1.66	1	94		−0.05		0.62	0.028	
	C3	Manage at school	4.22	0.82	1	−1.00	3	83		−0.04		0.55	0.503	
	C4	Social Activities	4.56	0.73	0	−1.60	2	90		−0.04		0.56	0.485	
	C5	Decision Making	4.12	0.85	0	−0.76	4	79		−0.02		0.50	0.739	
	C6	Support from Others	4.32	0.78	0	−0.96	3	86		−0.02		0.47	0.560	
	C7	Ability to Move	4.7	0.7	0	−2.87	2	93		−0.03		0.52	0.161	
Social Relationships									0.77		0.85			
	D1	Open up to Others	4.07	0.89	0	−0.81	5	77		−0.05		0.68	0.132	
	D2	Family Relationship	4.42	0.75	0	−1.05	1	87		0.00		0.42	0.152	
	D3	Relationship with Friends	4.45	0.71	0	−1.41	1	91		−0.05		0.65	0.039	
	D4	Friendships	4.53	0.69	0	−1.44	2	92		−0.03		0.57	0.103	
	D5	Attitudes of Others	4.22	0.76	0	−0.75	2	84		−0.04		0.63	0.359	
	D6	Demands from Others	4.01	0.84	0	−0.95	4	78		−0.04		0.60	0.080	
Emotions									0.73		0.76			
	E1	Loneliness	4.01	1.13	1	−0.89	13	70		0.01		0.46	0.028	
	E2	Anxiety	3.83	1.13	0	−0.81	13	67		−0.07		0.64	0.326	
	E3	Sadness	3.39	1.24	0	−0.44	24	53		−0.15		0.77	<0.001 *	0.016
	E4	Anger	3.41	1.25	0	−0.28	27	51		−0.04		0.57	0.230	
Physical Problems									0.71		0.83			
	F1	Clumsiness	3.55	1.21	0	−0.62	21	60		−0.01		0.44	0.028	
	F2	Other Injuries	4.13	1.24	0	−1.24	13	73		−0.05		0.57	0.007 *	0.014
	F3	Headaches	3.49	1.41	0	−0.58	26	60		−0.05		0.57	0.222	
	F4	Pain	3.74	1.15	0	−0.64	15	62		−0.05		0.58	0.601	
	F5	Seeing/Hearing	4.25	1.11	0	−1.41	11	80		−0.05		0.57	0.019	
	F6	TBI Effects	4.34	1.01	0	−1.57	7	81		−0.03		0.50	0.380	
Total Score									0.91		0.92			

Note: *M* = mean; *SD* = standard deviation; % = percent; *SK* = skewness. Negative values indicate left-skewed distributions. *R²* = McFadden’s R². *CITC* = corrected item–total correlations. Negative values in “Changes in α if Omitted” indicate a decrease in a scale’s Cronbach’s α if this item is omitted. *p* refers to a χ²-test between LORDIF models, including scale scores for an item only, to models including the scale score, the age category, and the age category and scale score interaction. McFadden’s R² is only reported for items with significant differences in model comparison. * = *p*-value < 0.01.

**Table 3:** Descriptive statistics of the QOLIBRI-KID/ADO, PedsQL, PHQ-9, GAD-7, and PCSI-SR8/13 questionnaires.

Instrument	Scoring	Scale	*N*	*M*	*SD*	*Min*	*Max*	*SK*
QOLIBRI-KID/ADO	0−100	Cognition	300	76.98	12.55	42.86	100.00	−0.47
		Self	298	77.63	15.24	15.00	100.00	−1.03
		Daily Life and Autonomy	300	86.85	11.18	42.86	100.00	−1.30
		Social Relationships	300	82.09	13.27	29.17	100.00	−0.98
		Emotions	300	66.47	22.00	6.25	100.00	−0.40
		Physical Problems (=Physical Functioning Score)	300	72.96	19.16	8.33	100.00	−0.83
		Psychosocial Functioning Score	298	75.82	12.51	26.82	100.00	−0.63
		Total Score	298	77.21	11.71	32.46	100.00	−0.68
PedsQL	0−100	Emotional Functioning	300	72.13	18.21	28.13	100.00	−0.73
		Social Functioning	299	88.23	12.53	0.00	100.00	−1.28
		School Functioning	299	76.90	15.34	40.00	100.00	−0.73
		Physical Functioning (= Physical Health Summary Score)	300	84.98	12.33	25.00	100.00	−1.39
		Psychosocial Health Summary Score	299	79.11	12.55	41.67	100.00	−0.77
		Total Score	299	81.15	11.60	36.96	100.00	−0.98
GAD-7	0−21	Total Score	299	3.77	3.30	0.00	17.00	1.87
PHQ-9	0−27	Total Score	299	3.39	3.19	0.00	22.00	1.44
PCSI-SR8	0−32	Total Score	165	5.48	4.92	0.00	25.00	1.46
PCSI-SR13	0−126	Total Score	132	20.37	18.64	0.00	82.00	1.05

Note: *N* = absolute frequencies; *M* = mean; *SD* = standard deviation; *Min* = minimum; *Max* = maximum; *SK* = skewness. QOLIBRI-KID/ADO = Quality Of Life after Brain Injury in Children and Adolescents (self-report); PedsQL = Pediatric Quality of Life Inventory (self-report); GAD-7 = Generalized Anxiety Disorder 7 (proxy report); PHQ-9 = Patient Health Questionnaire 9 (proxy report); PCSI-SR8/13 = Postconcussion Symptom Inventory (self-report).

**Table 4:** Test–retest reliability of the QOLIBRI-KID/ADO questionnaire for 57 participants after 10–20 days.

Scale	ICC(2,1)	SEm	MDC
Cognition	0.74 (0.59–0.84)	6.37	17.65
Self	0.76 (0.62–0.85)	7.03	19.50
Daily Life and Autonomy	0.62 (0.43–0.76)	5.68	15.74
Social Relationships	0.62 (0.44–0.76)	6.95	19.26
Emotions	0.67 (0.45–0.80)	12.59	34.91
Physical Problems	0.57 (0.33–0.73)	10.55	29.25
Total Score	0.78 (0.60–0.87)	4.82	13.35

Note: ICC(2,1) = intraclass correlation coefficient with 95% confidence interval; SEm = standard error of measurement; MDC = minimal detectable change.

**Table 5:** Results of known-group validity analyses of the QOLIBRI-KID/ADO questionnaire total score sociodemographic and clinical characteristics.

Known Groups	*n*	*M*	*SD*	*t*	*df*	*p*	*d*
Children	165	78.61	10.62	−2.32	296	0.01 *	−0.27
Adolescents	133	75.47	12.76				
Female	137	76.02	12.76	−2.26	296	0.01 *	−0.14
Male	161	78.61	10.96				
TBI within the last four years	100	76.19	11.89	−1.07	296	0.14	−0.13
TBI more than four years ago	200	77.72	11.61				
Moderate–severe TBI	60	76.67	12.24	−0.40	296	0.35	−0.06
Mild TBI	238	77.34	11.59				
Low Functional Recovery ^1^	15	-	-	-	-	-	-
High Functional Recovery ^1^	280	77.61	11.34				
Post-Concussion Symptoms ^2^	47	63.98	11.81	−10.03	293	<0.001 *	−1.60
No Post-Concussion Symptoms ^2^	248	79.92	9.62				
Head-/aches, Physical Impairments, or Mental Health Problems ^3^	146	74.93	11.67	−3.28	294	<0.001 *	−0.38
No Aches, Impairments, or Problems ^3^	150	79.33	11.41				
Low Learning Rate ^4^	57	77.73	12.99	0.41	295	0.66	0.06
Higher Learning Rate ^4^	240	77.02	11.38				
Mild to Severe Anxiety ^5^	87	72.14	13.07	−4.96	295	<0.001 *	−0.63
No Anxiety ^5^	210	79.28	10.46				
Mild to Severe Depression ^6^	88	71.46	12.74	−5.76	295	<0.001 *	−0.73
No Depression ^6^	209	79.61	10.39				

Note: *n* = sample size (only groups with n > 30 included in the analyses), *M* = mean, *SD* = standard deviation, *df* = degrees of freedom, *t* = t-value, *p* = p-value (* = *p* < 0.05) were adapted for one-tailed testing, *d* = Cohen’s *d*. ^1^ KOSCHI (low = 3a/b, 4a/b, high = 5a/5b; no further statistical analyses were performed for the KOSCHI due to the small sample of participants with low functional recovery; ^2^ PCSI-SR8/13 (symptoms = impaired: ≥ *M* + 1*SD*, not impaired ≤ *M*; ^3^ based on parent report; ^4^ RAVLT (low learning rate ≤ M – 1SD, not impaired: ≥ M); ^5^ GAD-7 (mild to severe ≥ 5, no < 5); ^6^ PHQ-9 (mild to severe ≥ 5, no < 5).

## Data Availability

The data presented in this study are available on request from the corresponding authors. The data are not publicly available for data protection reasons.

## Appendix A

**Table A1:** Changes in the wording of the German items in the pilot and final validation study translated into English.

	Wording *	
Item	Pilot Study	Final Validation Study
	How satisfied are you with…	
Orientation	… how you find your way around (for example, **at home or** how you can find the way to school)?	… how you **are able to** find your way around (for example, finding your way to school)?
Daily Independence	… being able to do **everyday things** without assistance (for example, using the toilet, getting dressed)?	… being able to do **things you do every day** without **help** (for example, getting dressed)?
Social Activities	KID: … how you are able to play with your friends **(for example, at school, at birthday parties)**? ADO: … how you are able **to join in with adolescents (for example, hobbies, parties, sports clubs)**?	… how you are able to play/**do things** with your friends?
Family Relationship	… how you get along with your family (parents, siblings)?	… how you get along with your family?

Note. * The English questionnaires were linguistically validated in the pilot and again for the changed items in the final validation study, therefore slight differences are possible in comparison to the German versions. The bold text refers to the removed/rephrased parts.

**Table A2:** Mean time in years since the TBIs of participants with and without neuropsychiatric symptoms.

Symptoms	Symptoms Present		Symptoms Absent		*t*-Test Parameters			
	*n*	*M* (*SD*)	*n*	*M* (*SD*)	*t*	*df*	*p*	*d*
Problems after TBI	147	5.06 (2.64)	151	5.71 (2.76)	2.09	296	0.04 *	0.24
Mild to Severe Anxiety	87	5.15 (2.66)	212	5.47 (2.74)	0.93	297	0.35	0.12
Mild to Severe Depression	89	4.99 (2.79)	210	5.55 (2.67)	1.64	297	0.10	0.21
Post-concussion Symptoms	47	5.13 (2.56)	250	5.41 (2.76)	−0.64	295	0.52	−0.10

Note: Post-concussion symptoms refer to a PCSI score one *SD* above the respective age average (see “Instrument” Section). *M* = mean time since the TBI in years; *SD* = standard deviation; *df* = degrees of freedom; *t* = *t*-value; *p* = *p*-value (* = *p* < 0.05); *d* = Cohen’s *d*.

**Table A3:** Internal consistency of the QOLIBRI-KID/ADO questionnaire scales for groups of participants stratified by sociodemographic and clinical characteristics.

Group	*n*	Cognition		Self		Daily Life and Autonomy		Social Relationships		Emotions		Physical Problems		Total Score	
		α	ω	α	ω	α	ω	α	ω	α	ω	α	ω	α	ω
Moderate/Severe TBI	60	0.68	0.76	0.71	0.82	0.76	0.84	0.80	0.86	0.64	0.84	0.69	0.81	0.91	0.93
Mild TBI	240	0.70	0.80	0.77	0.83	0.72	0.81	0.76	0.86	0.74	0.76	0.72	0.83	0.91	0.92
Problems Post-TBI ^1^	147	0.68	0.73	0.74	0.80	0.70	0.78	0.76	0.85	0.69	0.82	0.70	0.80	0.90	0.92
No Problems Post-TBI	151	0.69	0.78	0.78	0.83	0.75	0.80	0.78	0.85	0.77	0.83	0.69	0.80	0.91	0.93
Mild to Severe Anxiety	87	0.68	0.78	0.78	0.84	0.74	0.82	0.81	0.86	0.67	0.74	0.73	0.83	0.91	0.93
No Anxiety ^2^	212	0.66	0.75	0.73	0.80	0.70	0.80	0.72	0.82	0.74	0.80	0.68	0.80	0.89	0.91
Mild to Severe Depression	89	0.67	0.75	0.83	0.88	0.73	0.82	0.78	0.84	0.62	0.77	0.73	0.85	0.91	0.93
No Depression ^3^	210	0.65	0.75	0.69	0.81	0.70	0.80	0.75	0.84	0.76	0.82	0.68	0.81	0.89	0.91
Post-Concussion Symptoms ^4^	47	0.52	0.71	0.73	0.85	0.63	0.77	0.71	0.85	0.64	0.75	0.70	0.82	0.87	0.89
No Post-Concussion Symptoms	250	0.64	0.77	0.70	0.80	0.69	0.78	0.73	0.80	0.73	0.81	0.63	0.79	0.87	0.90
Low Learning Rate ^5^	57	0.75	0.85	0.82	0.89	0.79	0.84	0.78	0.84	0.65	0.76	0.75	0.87	0.93	0.95
High Learning Rate	242	0.67	0.76	0.74	0.80	0.71	0.79	0.77	0.86	0.74	0.79	0.71	0.83	0.90	0.92

Note: α = Cronbach’s Alpha coefficient, ω = McDonald’s Omega coefficient; ^1^ based on parent report; ^2^ GAD-7 (mild to severe ≥ 5, no < 5); ^3^ PHQ-9 (mild to severe ≥ 5, no < 5); ^4^ PCSI-SR8/13 (symptoms = impaired: *M* + 1*SD*, not impaired = *M* and *M* − 1*SD*); ^5^ RAVLT (low learning rate= *M* − 1*SD*, not impaired: *M* and *M* + 1*SD*).

**Table A4:** Correlations of the QOLIBRI-KID/ADO with the PedsQL, GAD-7, PHQ-9, and PCSI-SR8/SR13 questionnaires: convergent and divergent validity results (variance explained).

		PedsQL						PHQ-9	GAD-7	PCSI-SR8/SR13
		Emotional Functioning	Social Functioning	School Functioning	Physical Functioning	Psychosocial Functioning	Total Score	Total Score	Total Score	Total Score
**QOLIBRI-KID/ADO**	Cognition	20%	21%	39%	27%	39%	40%	18%	9%	26%
	Self	21%	11%	29%	22%	31%	32%	11%	5%	31%
	Daily Life and Autonomy	22%	26%	30%	34%	39%	43%	17%	8%	22%
	Social Relationships	24%	25%	27%	23%	38%	37%	10%	6%	18%
	Emotions	18%	9%	14%	14%	21%	21%	4%	3%	9%
	Physical Problems	14%	10%	24%	31%	23%	30%	10%	6%	20%
	Psychosocial Functioning	33%	23%	39%	32%	48%	49%	14%	8%	30%
	Total Score	34%	26%	44%	42%	52%	56%	17%	10%	34%

Note. The determination coefficient R^2^ is presented in percent (%); 0% = no variance explained, 100% = variance is perfectly explained. The shading represents a range from the lowest (yellowish cells) to the highest (greenish cells) amount of variance explained per row (i.e., from the perspective of the QOLIBRI-KID/ADO total and scale score).
